# Muscle wasting in osteoarthritis model induced by anterior cruciate ligament transection

**DOI:** 10.1371/journal.pone.0196682

**Published:** 2018-04-30

**Authors:** Jordana Miranda de Souza Silva, Paulo Vinicius Gil Alabarse, Vivian de Oliveira Nunes Teixeira, Eduarda Correa Freitas, Francine Hehn de Oliveira, Rafael Mendonça da Silva Chakr, Ricardo Machado Xavier

**Affiliations:** 1 Programa de Pós-Graduação em Medicina, Ciências Médicas, Universidade Federal do Rio Grande do Sul, Porto Alegre, RS, Brazil; 2 Laboratório de Doenças Autoimunes, Hospital de Clínicas de Porto Alegre, Porto Alegre, RS, Brazil; 3 Serviço de Patologia, Hospital de Clínicas de Porto Alegre, Porto Alegre, RS, Brazil; 4 Serviço de Reumatologia, Hospital de Clínicas de Porto Alegre, Porto Alegre, RS, Brazil; University of Memphis, UNITED STATES

## Abstract

This study aimed to investigate the molecular pathways involved in muscle wasting in an animal model of osteoarthritis (OA) induced by anterior cruciate ligament transection (ACLT) in rats. Reduction of protein syntheses, increased proteolysis and impaired muscle regeneration are important pathways related to muscle wasting, and myogenin, MyoD, myostatin and MuRF-1 are some of their markers. Female Wistar rats were allocated into two groups: OA (submitted to the ACLT) and SHAM (submitted to surgery without ACLT). Nociception, spontaneous exploratory locomotion and body weight of animals were evaluated weekly. Twelve weeks after the disease induction, animals were euthanized, and the right knee joints were collected. Gastrocnemius muscle of the right hind paw were dissected and weighed. Gastrocnemius was used for evaluation of muscle atrophy and expression of IL-1β, TNF-α, Pax7, myogenin, MyoD, myostatin and MuRF-1. Histopathology of the knee confirmed the development of the disease in animals of OA group. Gastrocnemius of OA animals showed a reduction of about 10% in area and an increased IL-1β expression compared to animals of SHAM group. Expression of myostatin was increased in OA group, while myogenin expression was decreased. TNF-α, Pax7, MuRF-1 and MyoD expression was similar in both OA and SHAM groups. Nociception was significantly elevated in OA animals in the last two weeks of experimental period. Spontaneous exploratory locomotion, body weight and weight of gastrocnemius showed no difference between OA and SHAM groups. Gastrocnemius atrophy in OA induced by ACLT involves elevated expression of IL-1β within the muscle, as well as increased expression of myostatin and decreased expression of myogenin. Therefore, muscle wasting may be linked to impaired muscle regeneration.

## Introduction

Osteoarthritis (OA) is a chronic disease characterized by abnormal remodeling of joint tissues driven by inflammatory mediators within the affected joint. The main sign of OA is the progressive degradation of articular cartilage, but changes in muscles surrounding the affected joint can also be present [1]. Since periarticular muscles function not only to produce movement, but also to provide joint stability, muscle impairment may influence the onset, progression and severity of OA [2]. Additionally, the presence of OA may have a negative impact on muscle function, further affecting the disease process, and establishing a vicious circle of joint degradation and muscle wasting [3].

In OA that affects lower limbs, muscle weakness is a common feature. Studies have identified the decline in muscle strength as an early phenomenon, and sometimes preceding structural lesions of the disease [4, 5]. Muscle weakness is also related with the progression of OA, since the muscle strength in the lower limbs seems to decrease as the severity of the disease increases [6–8]. Furthermore, muscle weakness in patients with OA is often associated with atrophy of muscle fibers [8, 9]. In this context studies report that there is a reduction of 12–19% in the cross-sectional area (CSA) of muscles from the affected limb in patients with hip and knee OA [9–11]).

In general, the main known molecular pathways leading to muscle wasting are related to reduction of protein syntheses, increased proteolysis and impaired muscle regeneration by satellite-cells [12]. Myostatin is recognized as a negative regulator of muscle mass growth and may promote protein degradation by stimulating the activity of ubiquitin-proteasome system [13]. The ubiquitin-proteasome system has skeletal muscle specific enzymes, such as MuRF-1 (Muscle Ring Finger-1) and MAFbx (Muscle Atrophy F-box), and plays a major role in muscle protein degradation [12]. Additionally, myostatin can impair the activation of satellite-cells reducing their capacity of proliferation and differentiation [14]. Satellite-cells are muscle stem cells that remain mitotically quiescent in adult muscle and, following an injury, they become activated and promote muscle growth and repair. Pax7 is a biomarker specifically expressed in all quiescent satellite-cells [15]. Once activated, satellite-cells proliferate, differentiate and then fuse to the existing myofibers for muscle regeneration. MyoD and myogenin are markers of satellite-cell proliferation and differentiation, respectively [15]. Finally, in addition to these pathways, as far as several pro-inflammatory cytokines, such as tumor necrosis factor-α (TNF-α) and interleukin‑1β (IL-1β), are related to elevated catabolism in inflammatory diseases, they may also play a role in the triggering of muscle wasting [16, 17].

Despite the knowledge about muscle weakness and atrophy in patients with OA of lower limbs, literature lacks basic science studies concerning the molecular processes involved in OA muscle wasting. Our aim was to identify which pathways are associated with muscle wasting in an animal model of OA induced by anterior cruciate ligament transection (ACLT). Our analyses demonstrated that, in ACLT model, muscle atrophy is related with increased levels of IL-1β and myostatin and decreased levels of myogenin within the muscle.

## Materials and methods

### Animals and study design

All procedures were conducted in accordance with the norms established by the Brazilian Animal Protection Law, normative resolutions of the National Council on the Control of Animal Experimentation (CONCEA), and were approved by Hospital de Clínicas de Porto Alegre Ethics Committee on the Use of Animals (CEUA) (Permit Number: 12–0040). All surgery was performed under isoflurane anesthesia, and all efforts were made to minimize suffering.

Three-month-old female Wistar rats weighing about 250g were studied. Animals were housed in plastic cages under controlled environmental conditions with free access to water and food. Eighteen rats were randomly distributed in two experimental groups of nine animals each: OA (submitted to the ACLT) and SHAM (submitted to surgical procedures without ACLT). Sample size was determined based on data from a pilot study, with reduced myofiber cross-sectional area as the primary outcome. Considering alpha value of 0.05 and power of 80%, the sample size was calculated as eight animals per group. Regarding a possible loss during the surgical procedures or along the experimental period, a margin of 10% was determined and, therefore, nine animals were allocated in each group.

### Induction of osteoarthritis

OA was induced by surgical transection of the right anterior cruciate ligament. Animals were initially anesthetized with an intraperitoneal injection of ketamine (0.50mg/kg) and xylazine (0.25mg/kg). During the surgical procedures, anesthesia was maintained with inhaled isoflurane (3%). Under anesthesia, the right knee was shaved and prepared using an iodine solution and a 3cm incision was made medial to the patellar tendon. The subcutaneous tissue and muscle were then incised and the patella laterally sublaxed; the joint capsule was opened with the limb hyperextended. With the limb in full flexion, the anterior cruciate ligament was visualized by blunt dissection, and sectioned by a latero-medial cut parallel to the tibial plateu, using a scapel blade. Transection was confirmed with the anterior drawer test. The patella was then replaced, and the limb extended. The joint capsule and muscle layers were closed with 5.0 Vicryl sutures and 50μL of tramadol was then injected into the joint capsule to provide local analgesia. Skin was closed with 4.0 nylon sutures. Sham surgery consisted of all the steps mentioned the above except for ligament transection. After surgery, the animals were returned to their cages and received intraperitoneal injection of tramadol (5mg/kg). Twelve and 24 hours after the surgery, the animals received tramadol intraperitoneally (5mg/kg) as postoperative systemic analgesia.

### Experimental period

The experimental period lasted 12 weeks. In the first two weeks after surgery, the animals were kept at rest. After the third week post-surgery, animals were weighed and had their spontaneous exploratory locomotion evaluated weekly. At the end of experimental period, at the twelfth week, animals were anesthetized with inhaled isoflurane and then euthanized by decapitation. The right knee joint was removed to confirm the development of OA through histopathological analysis. Gastrocnemius muscle was isolated, dissected and weighed. The muscles were stored at -80°C and were used for histological analysis, immunohistochemistry and Western blot.

### Animal locomotion

The locomotion of animals was evaluated before the surgery and, after the third week post-surgery, it was evaluated weekly until the end of the experimental period. In this procedure, the rats were placed individually into an acrylic box, with sensors capable of detecting the motion of animals (Monitor de Atividade IR, Insight Equipamentos Ltda, Ribeirão Preto, SP, Brazil). In each evaluation, spontaneous exploratory locomotion of the animals was detected for 5 minutes after an adaptation period of 30 seconds. Data from the motion detection were sent to and evaluated by a software (Insight Equipamentos Ltda, Ribeirão Preto, SP, Brazil) using the following parameters: walked distance and velocity.

### Nociception

The nociception of animals was evaluated before the surgery and, after the third week post-surgery, it was evaluated weekly until the end of the experimental period. The nociceptive mechanical threshold from the right hind paw was measured by the electronic Von Frey method (electronic Von Frey, Insight Equipamentos Ltda, Ribeirão Preto, SP, Brazil). Mice were placed in acrylic cages (12 x 20 x 17 cm) with wire grid floors in a quiet room 30 minutes before the experiment. The test consisted of evoking a hind paw flexion reflex with a hand-held force transducer adapted with a tip. The investigator was trained to apply the tip in the plantar region with a gradual increase in pressure. The stimulus was automatically discontinued, and its intensity was recorded, in grams (g), when the paw was withdrawn.

### Knee joint histopathology

The joints from the right knee were excised and fixed in 10% buffered formalin for five days. The joints were decalcified with 10% nitric acid for 24h and paraffin mounted. The joints were sectioned into 3 μm slices and stained with Safranin O fast green. Two different scoring systems developed by OARSI (Osteoarthritis Research Society International) were used to evaluate the animal joints and to measure the experimental OA severity by one blinded pathologist: OARSI histopathology grading system and OARSI cartilage degeneration score.

The OARSI histopathology grading system consists of OA cartilage pathology assessment system based on six grades, which reflect depth of the lesion, and four stages reflecting extent of OA over the joint surface. The recommended score is an index of combined grade and stage. The simple formula: score = grade x stage is recommended. This method produces an OA score with a range of 0–24 based on the most advanced grade and most extensive stage present [18].

The OARSI cartilage degeneration score is an evaluation of overall cartilage pathology. For this score, the medial tibial cartilage plateau is divided into three zones in order to evaluate pathology of different load-bearing areas. Cartilage degeneration in each zone is scored 0–5, according to the percentage of affected cartilage [19].

### Myofiber cross-sectional area

Myofiber cross-sectional area measurement was used to evaluate muscle atrophy. The gastrocnemius muscles from the right hind limbs were excised and fixed in 10% buffered formalin for 1 day and paraffin mounted. The muscles were sectioned into 6 μm slices and stained with hematoxylin eosin (HE). To determine the myofiber area, the muscle fiber diameter was measured, and the myofiber CSA was calculated. Using the software Image-Pro Express (version 5.1.0.12; Media Cybernetics, Rockville, MD, USA), 10 images were taken of each gastrocnemius muscle per rat, and 20 fibers were measured from each image, totaling 200 measured myofibers [20].

### Immunohistochemistry

To verify the inflammatory grade and the content of quiescent satellite-cells within the muscle, gastrocnemius of ten animals (five animals of OA group and four animals of SHAM group) were used for detection of IL-1β, TNF-α and Pax7 expression by way of immunohistochemistry. After fixation with 10% neutral buffered formalin, paraffin-embedded gastrocnemius slices were sectioned at 3 μm. Sections were deparaffinized in xylene, rehydrated, washed in distilled water and phosphate-buffered saline (PBS) and immersed in citrate buffer with pH 6.0. The sections were incubated in 5% hydrogen peroxide in methanol to reduce endogenous peroxidase activity and then in 5% powdered skimmed milk diluted in PBS to block nonspecific staining. Subsequently, sections were incubated with primary antibody against IL-1β (1:50; Santa Cruz Biotechnology Inc, sc-1251), TNF-α (1:50; Santa Cruz Biotechnology Inc, sc-1349) and Pax7 (1:200; Abcam, ab34360) at 4°C overnight. After incubation, immunodetection was performed with secondary antibody against goat immunoglobulin G (1:200, Sigma, A5420) or against rabbit immunoglobulin G (1:200, Sigma, A9169), followed by peroxidase labelled streptavidin and diaminobenzidine chromogen as substrate (Dako, K3468). The sections were counterstained with hematoxylin. All sections were analyzed by ImageJ software (ImageJ v1.43j; National Institutes of Health, Bethesda, MD, available at http://rsbweb.nih.gov/ij/) to measure the integrated optical density (IOD) on the images amplified by 200-fold. The following formula was applied: IOD = LOG (max intensity/mean intensity); for “max intensity”, it was assumed 255 for 8 bit images.

### Western blot

Western blot was performed to identify the protein expression of myogenin, MyoD, myostatin and MuRF-1. The muscle samples were homogenized with a lysis buffer (10mM Tris-HCl, 250mM saccharose, 5mM EDTA, 50mM sodium chloride, 30mM anhydrous sodium phosphate, 50mM sodium fluoride, 100μM sodium orthovanadate, 10mM phenylmethylsulfonyfluoride (PMSF), 100mM Dithiothreitol (DTT), and protease inhibitor cocktail in MiliQ water). Homogenates were centrifuged at 12.000 g for 10 minutes at 4°C and the supernatant was used. Protein concentration was determined by Bradford assay. Muscle proteins (100μg) were separated on 12% sodium dodecyl sulfate-polyacrylamide gel electrophoresis and transferred to a polyvinylidene difluoride (PVDF) membrane. The membranes were stained with Ponceau solution to confirm the protein transfer and then rinsed with phosphate-buffered saline with Tween (PBS-T). The membranes were incubated under low agitation overnight at 4°C with the primary antibodies against myogenin (1:250, Sigma, SAB2501587), MyoD (1:500, Sigma, SAB2501587), myostatin (1:250, Santa Cruz, sc-28910), MuRF-1 (1:500, Abcam, ab-172479), and diluted in milk/PBS-T (1% powdered skimmed milk). After the primary antibody incubation period, the membranes were washed for 30 minutes in PBS-T, incubated with secondary antibody against rabbit immunoglobulin G (1:5000, Sigma, A9169) or against goat immunoglobulin G (1:5000, Sigma, A5420) diluted in milk/PBS-T (1% powdered skimmed milk) for 2 hours at room temperature and then washed again for 30 minutes in PBS-T. Detection of the labeled protein was done using the enhanced chemiluminescence system (Millipore, WBKLS0500). These protein expressions were normalized by GAPDH expression.

### Statistical analyses

Statistical analyses were performed using SPSS 21.0 (Armonk, New York, USA). First, Shapiro-Wilk normality test was performed. *In vivo* data, such as spontaneous exploratory locomotion, body weight and nociception, along the 12 weeks of experimental period, were analyzed by generalized estimating equation (GEE) method and these results are expressed as mean and standard error. The remaining data were not normally distributed and Mann–Whitney's *U*-test was performed; these results are expressed as medians and interquartile range. Significance was accepted at p < 0.05.

## Results

Among the 18 rats, 1 rat from SHAM group died during the anesthesia procedure before the surgery. Data for this rat were collected at pre-surgery only.

### Animal locomotion

**Fig 1:**
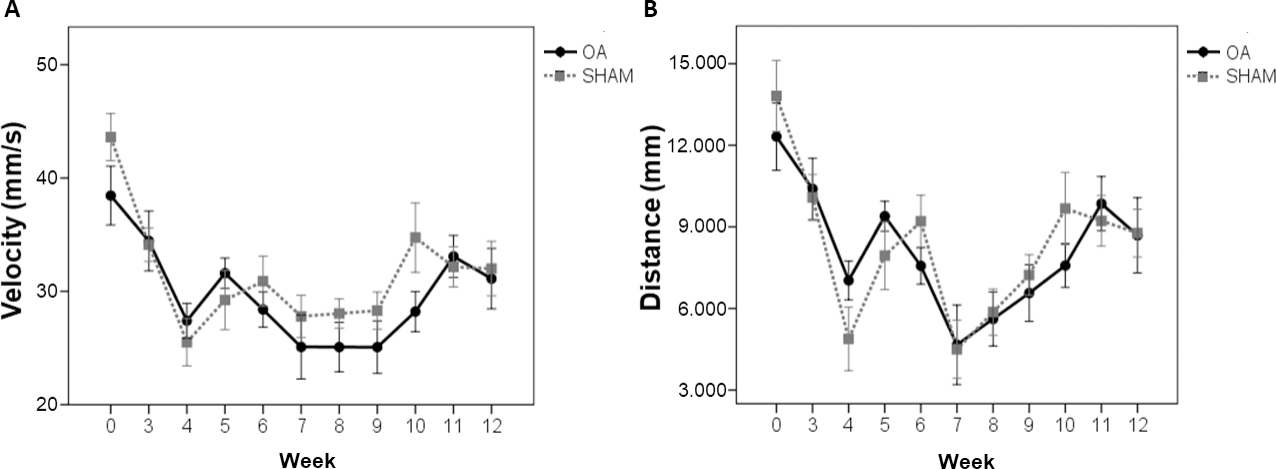
Spontaneous exploratory locomotion was not different between groups. (A) Velocity and (B) walking distance of OA and SHAM animals during the experimental period. Data are expressed as mean ± SEM.

During the twelve weeks of the experimental period, none of the parameters of spontaneous exploratory locomotion (velocity and walked distance) showed significant difference between OA and SHAM groups (Fig 1A and 1B).

### Nociception

**Fig 2:**
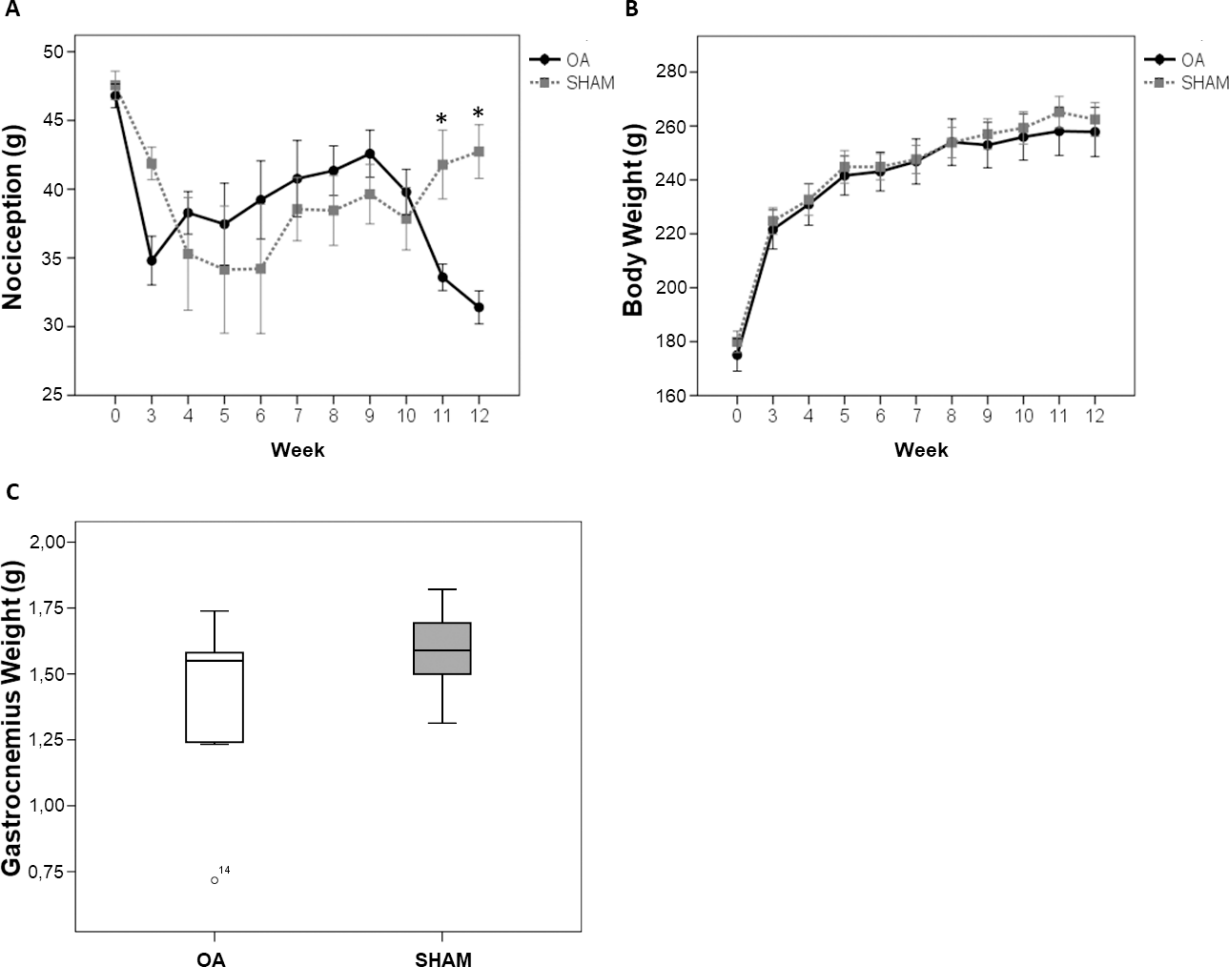
OA increased nociception at weeks 11 and 12, and did not change body and gastrocnemius weight. (A) Nociception and (B) body weight of OA and SHAM animals during the experimental period. Data are expressed as mean ± SEM. (C) Weight of right hind paw gastrocnemius of OA and SHAM animals at the end of the experimental period. Data are expressed as medians with interquartile range.

The nociception of the right hind paw was significantly increased in animals of OA group in the last two weeks of the experimental period, compared to animals from SHAM group (p = 0.0011; Fig 2A).

### Animal and muscle weight

Body weight of the animals in both groups increased equally over the experimental period, therefore there was no statistical difference in body weight between OA and SHAM groups, during the experimental period (Fig 2B). Regarding the weight of the right hind paw muscle, the median (25,75 percentile) of gastrocnemius weight was lower in OA group 1,55g (1,24, 1,58) compared to SHAM group 1,59g (1,51, 1,66), but not significantly different (Fig 2C) at the end of the experimental period.

### Knee joint histopathology

**Fig 3:**
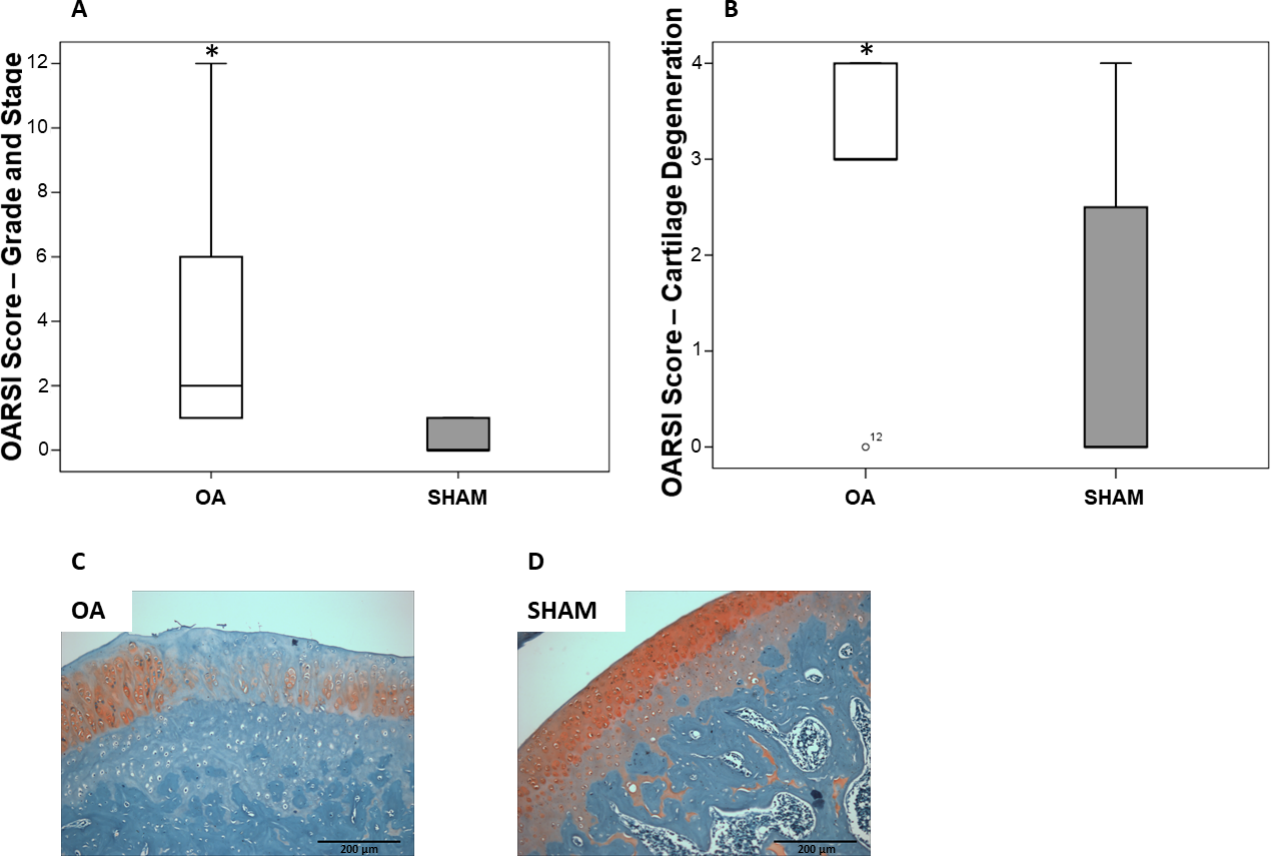
Histopathological evaluation of the knee joints showed osteoarthritis development. (A) OARSI grading system score and (B) OARSI cartilage degeneration score of OA and SHAM animals. Representative samples of Safranin O stained right knee joint of (C) OA and (D) SHAM animals at the end of the experimental period. Magnification: × 200. Data are expressed as medians with interquartile range. *p < 0.05 OA vs SHAM.

At 12 weeks after ACL transection, in the knee joint of animals from OA group, most of the remaining cartilage was degraded and undergoing remodeling. There was cartilage replacement by fibrous connective tissue in animals from OA group, compromising cartilage original function. Moreover, synovial hyperplasia and bone remodeling were also detected. Statistical difference between OA and SHAM groups was found in both OARSI grading system (p = 0.0029; Fig 3A) and OARSI cartilage degeneration score (p = 0.0304; Fig 3B).

### Muscle cross-sectional area

**Fig 4:**
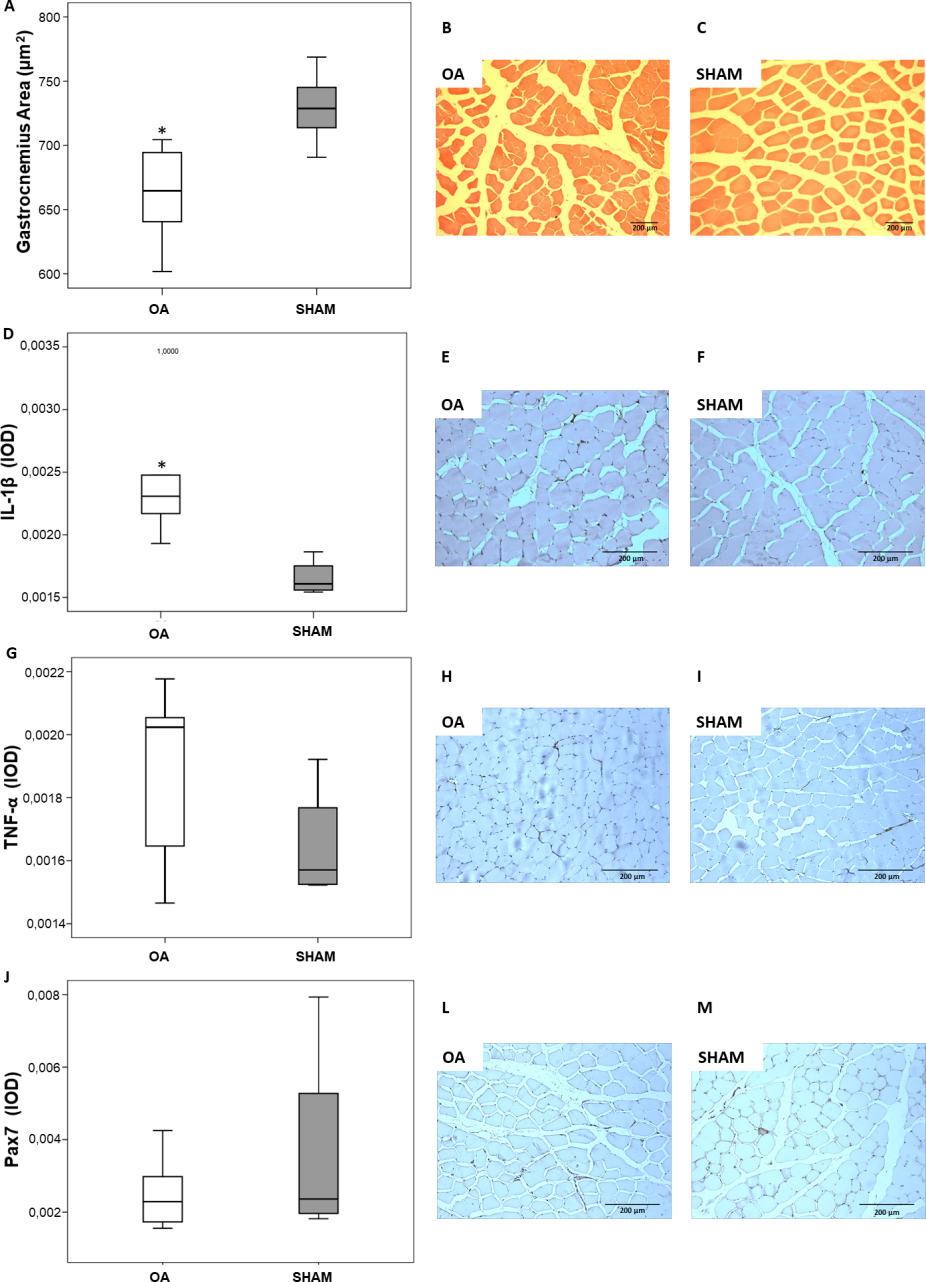
OA animals had decreased gastrocnemius area, and increased IL-1β expression. TNF-α and Pax7 expressions were unchanged. (A) Gastrocnemius CSA and representative samples of right hind paw gastrocnemius stained with hematoxylin-eosin of (B) OA and (C) SHAM animals at the end of the experimental period. (D) IL-1β expression estimated as IOD and representative samples of right hind paw gastrocnemius stained for IL-1β of (E) OA and (F) SHAM animals at the end of the experimental period. (G) TNF-α expression estimated as IOD and representative samples of right hind paw gastrocnemius stained for TNF-α of (H) OA and (I) SHAM animals at the end of the experimental period. (J) Pax7 expression estimated as IOD and representative samples of right hind paw gastrocnemius stained for Pax7 of (L) OA and (M) SHAM animals at the end of the experimental period. Magnification: × 200. Data are expressed as medians with interquartile range. *p < 0.05 OA vs SHAM.

The evaluation of gastrocnemius CSA, after 12 weeks of disease, demonstrated that animals form OA group presented muscle atrophy. A reduction of approximately 10% in gastrocnemius CSA was found in animals from OA group, in comparison with animals from SHAM group (p = 0.0006; Fig 4A).

### Immunohistochemistry

Immunostaining for IL-1β, TNF-α and Pax7 was done on paraffin sections of gastrocnemius muscle obtained from animals of OA and SHAM groups. At 12 weeks post-surgery, IL-1β expression estimated as IOD was significantly stronger in animals of OA group compared to animals of SHAM group (p = 0,0392; Fig 4B). Expression of TNF-α and Pax7 was not different between OA and SHAM groups.

### Western blot

**Fig 5:**
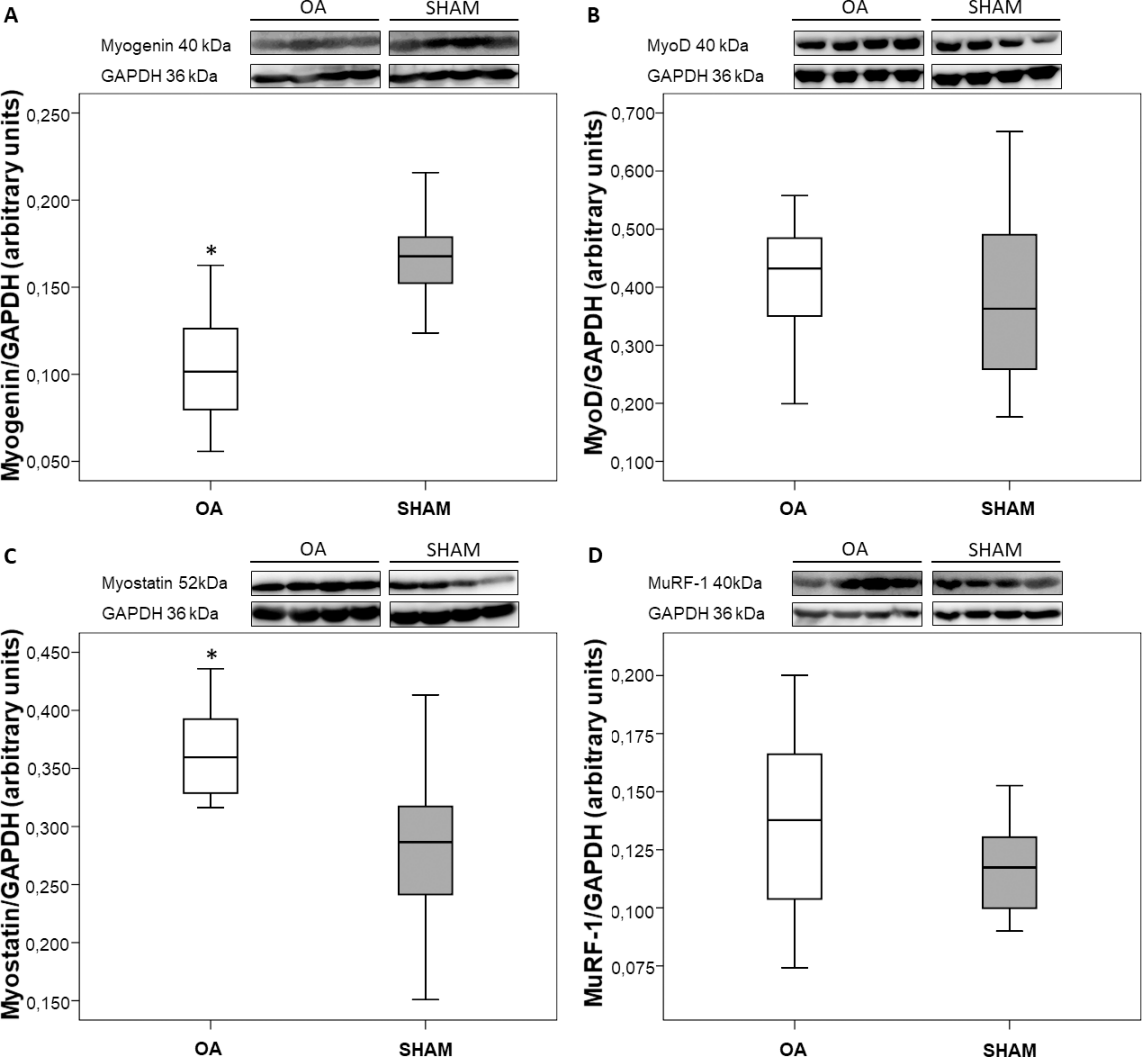
Myogenin expression was decreased, and myostatin expression was increased in OA animals. Western blot analyses showing the expression of (A) myogenin, (B) MyoD, (C) myostatin and (D) MuRF-1 in gastrocnemius muscle of OA and SHAM animals at the end of the experimental period. Data are expressed as medians with interquartile range. *p < 0.05 OA vs SHAM.

After 12 weeks of disease, gastrocnemius expression of myogenin (satellite-cell differentiation marker) displayed significantly lower levels in OA group (0.4 fold, p = 0.0011; Fig 5A), while MyoD (satellite-cell proliferation marker) expression did not show difference between groups at the end of the experimental period (Fig 5B).

Additionally, increased expression of myostatin (negative regulator of muscle mass) protein (52kDa; larger myostatin propeptide) was found in gastrocnemius muscles from OA group (0.3 fold, p = 0.0184; Fig 5C). Mature myostatin peptide (26kDa) was not detected. The primary antibody used was supposed to detect both 52 and 26kDa proteins, but only 52kDa protein was detected.

The expression of MuRF-1 (ubiquitin-proteasome marker) did not differ between OA and SHAM groups (Fig 5D).

## Discussion

The results of this study provide new information about the molecular pathways involved in muscle wasting in a rat model of OA induced by ACLT. The muscle atrophy found in animals of OA group seems to be representative of what occurs in patients with OA. Additionally, the increased levels of IL-1β and myostatin and the decreased levels of myogenin, in gastrocnemius muscle of OA animals, may partially explain the mechanisms involved in this atrophy.

An experimental period of 12 weeks was established, since, at this time, ACLT model is able to promote a significant joint cartilage damage [21]. Moreover, after 12 weeks of disease, muscle atrophy was also expected to be present, allowing the evaluation of pathways involved in muscle wasting. To verify the occurrence and the severity of the disease, two different scoring systems developed by OARSI were used to evaluate joint histopathology: OARSI histopathology grading system and OARSI cartilage degeneration score. The first system is a combined score based on qualitative assignment of numbers as OA histologic features, while the second one is a measurement of cartilage degeneration percentage. These scores complement each other, and both showed significant difference between OA and SHAM groups. According to OARSI grading system, most animals of OA group developed a mild disease, while according to OARSI cartilage degeneration score, animals of OA group had severe cartilage degeneration, but the animals from SHAM group also had a small degree of joint involvement, probably due to sham surgery.

Because of joint impairment, patients with OA typically suffer from joint pain and its consequences such as avoidance of physical activity. In the long-term, the avoidance of activities, to prevent pain, leads to muscle strength deterioration [22, 23]. In our study, animals of OA group showed significant increase in nociception only in the last two weeks of the experimental period. Regarding to the spontaneous exploratory locomotion test, the animals of OA group had the same pattern of movement than animals of SHAM group throughout the experimental period. Based on these results, it is likely that the muscle wasting seen in our model may not be closely associated with disuse. Otherwise, as OA animals exhibited a significant increase in nociception in the end of the study, it is possible that a reduction in activity levels was detected if the experimental period was longer.

Regarding body weight, animals from both groups gained weight at the same rate throughout the experimental period. The weight of gastrocnemius also showed no significant difference between groups. These results demonstrate that OA induced by ACL transection is unable to promote a marked loss of lean body mass in animals of OA group. Differently, in other models in which animals develop muscle atrophy, such as models of immobilization or chronic inflammation, there is a significant loss of body weight and loss of muscle weight [24, 25]. In our model, the lack of loss of muscle mass in animals of OA group comparing to SHAM group agrees with the mild atrophy that OA animals developed.

At the end of the experimental period, gastrocnemius area of OA animals had a reduction of about 10% compared to animals of SHAM group. The atrophy that occurred in these animals was mild, but it demonstrates that OA induced by ACL transection can promote muscle impairment. The absence of reduction in levels of locomotion in animals of OA group shows that muscle atrophy may be related to other factors and not solely to disuse. As the immobility does not seem to be the only cause of muscle atrophy in our model, muscle wasting may also be associated with the chronic inflammatory process of the disease.

It is noteworthy that OA is considered a low-grade inflammation disease, mainly because of synovitis [26]. Serum concentration of several inflammatory markers is elevated in patients with OA of the lower limbs, and it is correlated with decreased physical function and lower muscle strength [27, 28]. Levels of inflammatory mediators are also increased in muscles of patients with advanced knee OA, when compared to healthy individuals, and are related with physical disability and reduced muscle strength [3, 29]. We reported higher levels of IL-1β in gastrocnemius muscles of animals with OA, while TNF-α levels were not significantly increased in OA group. After twelve weeks of experimental period, OA induced by ACLT significantly altered IL-1β expression in periarticular muscles.

In many pathologies, muscle wasting is associated with chronically elevated circulating levels of inflammatory cytokines [16, 17]. Accordingly, animal models of cancer cachexia and sepsis demonstrated that there is a concomitant imbalance in myofibrillar protein synthesis and proteolysis [30, 31]. Otherwise, the myogenic stem cells, called satellite-cells, give the adult skeletal muscle the ability to regenerate in response to muscle damage. During the quiescence, satellite cells are characterized by their expression of Pax7. Due to stimuli, satellite-cells become activated and co-express Pax7 and MyoD. Most of the activated satellite-cells proliferate, then downregulate Pax7 and start differentiation. Afterwards, during differentiation, MyoD expression declines due to activation of myogenin [15]. Among growth factors that are secreted during muscle repair, insulin-like growth factors (IGFs) play a pivotal role, influencing muscle cell proliferation and differentiation, as well as muscle hypertrophy [32].

Increased levels of TNF-α or IL-1β have been shown to inhibit the expression and activity of hormones such as IGFs. In vitro experiments demonstrate that inflammatory cytokines act directly on myoblasts by impairing the ability of IGF-I to promote their differentiation into more mature cells [33, 34]. Specifically, IL-1β impairs IGF-I-induced expression of myogenin [33]. Our results show a decrease in myogenin protein expression in animals of OA group, although MyoD levels remained the same in both OA and SHAM groups. It is remarkable that no other study has ever reported on markers of satellite-cell activation in OA-related muscle wasting. According to the literature, gene and protein expression of these markers are usually decreased in disuse atrophy, as well as in inflammatory diseases [24, 35–39]. Myogenin is necessary for efficient activation of target genes required for terminal differentiation of myoblasts, and further fusion of myoblasts to the existing myofibers for muscle repair [40]. Furthermore, in the absence of myogenin, other muscle regulatory factors, such as MyoD, cannot promote muscle formation, since the differentiation of satellite-cells is impaired [41]. As the expression of Pax7 is not altered and, therefore, there is no reduction in the total amount of satellite-cells in OA animals, the decreased satellite-cell differentiation may be the main contributor to muscle atrophy in this model.

Additionally, binding of TNF-α and IL-1β to their respective receptors can trigger pathways that result in the activation of nuclear factor kappa B (NF-κB) [22]. When activated, NF-κB translocates to the nucleus and promotes the transcription of a number of cytokine and chemokine, growth regulatory, and survival genes. It has been demonstrated that increase in TNF-𝛼 stimulates NF-𝜅B associated with upregulation of myostatin.

Myostatin protein is produced in muscle and adipose tissue and then is released and circulates in the blood in a latent form as a full-length precursor, which is cleaved into an amino-terminal pro-peptide and a carboxy-terminal mature region: the active form of the molecule [42]). After twelve weeks of disease, increased levels of myostatin protein (52kDa; larger myostatin propeptide) were found in animals of OA group. Mature myostatin peptide (26kDa) was not detected. Findings from several groups indicate that myostatin expression is usually increased during conditions such as cachexia [35, 43–45]). In disuse conditions, however, levels of myostatin are usually decreased or not changed [46, 47]. Moreover, myostatin inhibition is able to increase muscle mass and prevent loss of muscle mass in several pathological conditions in mice [48, 49]. So, the elevation of myostatin expression in animals of OA group may be a contributing factor to the atrophy that was found in these animals. The only study that evaluated the expression of myostatin in muscles of patients with hip OA reported increased levels of myostatin gene expression, which is in accordance with our results [50].

As far as myostatin can stimulate protein degradation by activating forkhead box O (FoxO) pathway, muscle proteolysis induced by ubiquitin-proteasome system was expected to be enhanced in animals of OA group. However, although OA animals presented higher expression of MuRF-1, this increase was not significant compared to SHAM group. On the other hand, it is worth mentioning that OA animals developed a mild muscle atrophy (reduction of about 10% in myofiber area), which may be in accordance with the absence of increased MuRF-1 expression in these animals. In other conditions, such as different types of cachexia (cancer, heart failure, chronic obstructive pulmonary disease) and in response to disuse, levels of ubiquitin-proteasome markers are usually increased [13, 51, 52].

Myostatin also plays a negative role in the control of satellite-cell proliferation and differentiation and MyoD and myogenin are implicated to participate in myostatin-induced differentiation suppression [14, 53]. In animals of OA group, higher levels of myostatin might also be related to the repression of the satellite-cell differentiation program.

According to our results, higher levels of muscle IL-1β are likely related to the increase in myostatin expression and the reduction of satellite-cell differentiation by lower myogenin expression. As inflammatory mediators and myostatin interfere with the function of satellite-cells, by decreasing or blocking their ability to fuse with or replace damaged myofibers, their action may ultimately result in muscle wasting. Therefore, the expression pattern of muscle markers, added to the absence of changes in locomotion in our model of post-traumatic OA, demonstrates that, at least initially, the OA-related muscle atrophy is associated with the process of the disease. Nevertheless, with advancement of the disease and the worsening of joint impairment and pain, atrophy shall be related not only to chronic inflammation, but also to disuse.

As limitations of the study, OA induced by ACLT produces a mild disease, and a mild muscle atrophy. Moreover, the animals were up to 24 weeks of age and the disease development was confirmed by the evaluation of only one pathologist. Despite being a mild model, it resembles the OA that occurs in humans, which is usually a low-grade inflammation disease. Additionally, the sample size may have influenced the non-normality of the data.

## Conclusion

This study provided new insights on the molecular mechanisms involved in muscle wasting in OA induced by ACLT. The pathways involved could be partially identified and, from now, it is known that the inflammatory process of the disease and the impaired muscle regeneration play a significant role in OA-related muscle wasting. This knowledge can be useful for assessing the effects of rehabilitation interventions and for prevention strategies against the disease development.

## Supporting information

S1 Fig All western blot images of OA and SHAM animals. Western blot analyses showing the expression of myogenin, MyoD, myostatin and MuRF-1 in gastrocnemius muscle of OA and SHAM animals at the end of the experimental period.
(TIF)

S2 Fig All safranin O stained samples of OA and SHAM animals. Right knee joint slides of OA and SHAM animals at the end of the experimental period. Magnification: × 200.
(TIF)

S3 Fig Gastrocnemius images of animals 1, 2 and 3. Samples of right hind paw gastrocnemius stained with hematoxylin-eosin of OA and SHAM animals at the end of the experimental period. Magnification: × 200.
(TIF)

S4 Fig Gastrocnemius images of animals 4, 5 and 6. Samples of right hind paw gastrocnemius stained with hematoxylin-eosin of OA and SHAM animals at the end of the experimental period. Magnification: × 200.
(TIF)

S5 Fig Gastrocnemius images of animals 7, 9 and 10. Samples of right hind paw gastrocnemius stained with hematoxylin-eosin of OA and SHAM animals at the end of the experimental period. Magnification: × 200.
(TIF)

S6 Fig Gastrocnemius images of animals 11, 12 and 13. Samples of right hind paw gastrocnemius stained with hematoxylin-eosin of OA and SHAM animals at the end of the experimental period. Magnification: × 200.
(TIF)

S7 Fig Gastrocnemius images of animals 14, 15 and 16. Samples of right hind paw gastrocnemius stained with hematoxylin-eosin of OA and SHAM animals at the end of the experimental period. Magnification: × 200.
(TIF)

S8 Fig Gastrocnemius images of animals 17 and 18. Samples of right hind paw gastrocnemius stained with hematoxylin-eosin of OA and SHAM animals at the end of the experimental period. Magnification: × 200.
(TIF)

S9 Fig IL-1 β immunohistochemistry images of animals 1, 2 and 3. Samples of right hind paw gastrocnemius of OA and SHAM animals stained for IL-1β, at the end of the experimental period. Magnification: × 200.
(TIF)

S10 Fig IL-1 β immunohistochemistry images of animals 4, 5 and 6. Samples of right hind paw gastrocnemius of OA and SHAM animals stained for IL-1β, at the end of the experimental period. Magnification: × 200.
(TIF)

S11 Fig IL-1 β immunohistochemistry images of animals 7, 9 and 10. Samples of right hind paw gastrocnemius of OA and SHAM animals stained for IL-1β, at the end of the experimental period. Magnification: × 200.
(TIF)

S12 Fig TNF-α immunohistochemistry images of animals 1, 2 and 3. Samples of right hind paw gastrocnemius of OA and SHAM animals stained for TNF-α, at the end of the experimental period. Magnification: × 200.
(TIF)

S13 Fig TNF-α immunohistochemistry images of animals 4, 5 and 6. Samples of right hind paw gastrocnemius of OA and SHAM animals stained for TNF-α, at the end of the experimental period. Magnification: × 200.
(TIF)

S14 Fig TNF-α immunohistochemistry images of animals 7, 9 and 10. Samples of right hind paw gastrocnemius of OA and SHAM animals stained for TNF-α, at the end of the experimental period. Magnification: × 200.
(TIF)

S15 Fig Pax7 immunohistochemistry images of animals 1, 2 and 3. Samples of right hind paw gastrocnemius of OA and SHAM animals stained for Pax7, at the end of the experimental period. Magnification: × 200.
(TIF)

S16 Fig Pax7 immunohistochemistry images of animals 4, 5 and 6. Samples of right hind paw gastrocnemius of OA and SHAM animals stained for Pax7, at the end of the experimental period. Magnification: × 200.
(TIF)

S17 Fig Pax7 immunohistochemistry images of animals 7, 9 and 10. Samples of right hind paw gastrocnemius of OA and SHAM animals stained for Pax7, at the end of the experimental period. Magnification: × 200.
(TIF)
