# Supplementary figures and images for: Muscle wasting in osteoarthritis model induced by anterior cruciate ligament transection
Source: PLoS One. 2018 Apr 30;13(4):e0196682. doi: 10.1371/journal.pone.0196682 (PMC5927423; doi:10.1371/journal.pone.0196682)

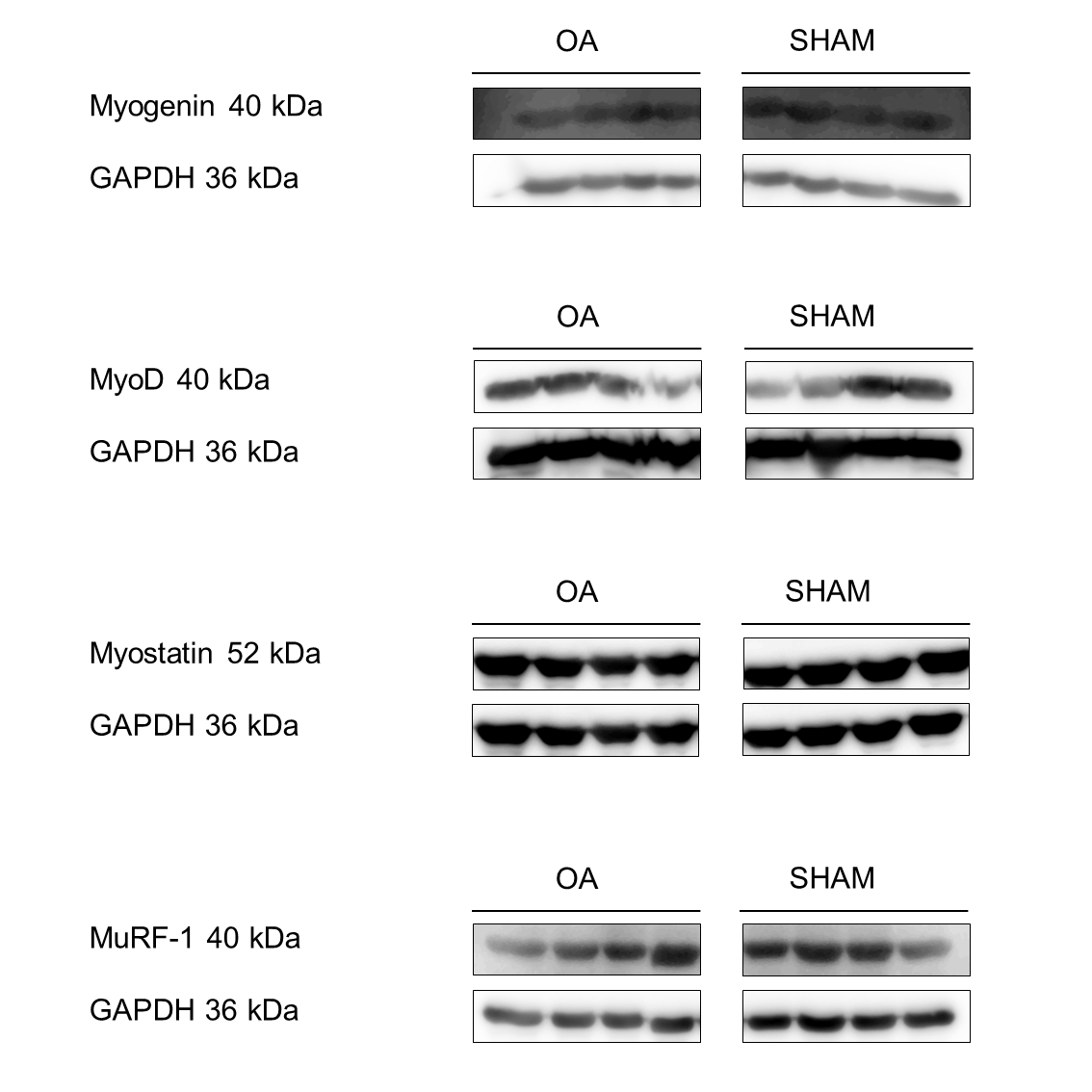

Supplement: S1 Fig — Western blot analyses showing the expression of myogenin, MyoD, myostatin and MuRF-1 in gastrocnemius muscle of OA and SHAM animals at the end of the experimental period. (TIF) [file pone.0196682.s001.tif]

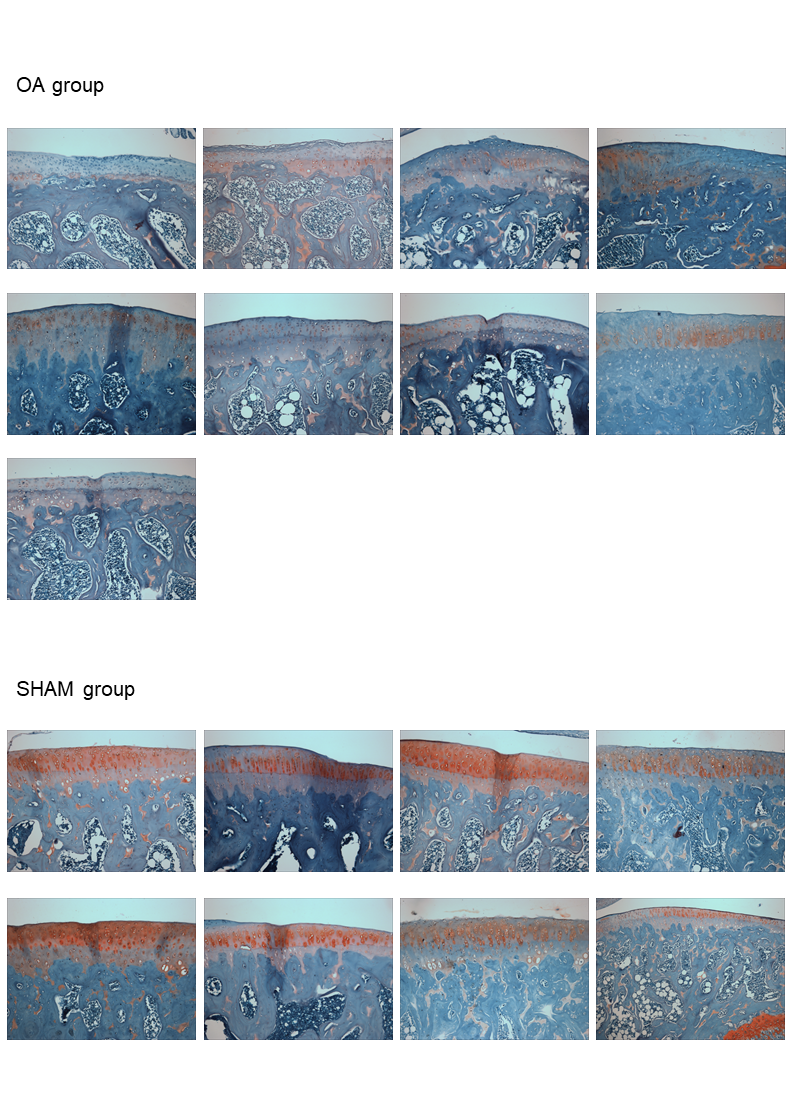

Supplement: S2 Fig — Right knee joint slides of OA and SHAM animals at the end of the experimental period. Magnification: × 200. (TIF) [file pone.0196682.s002.tif]

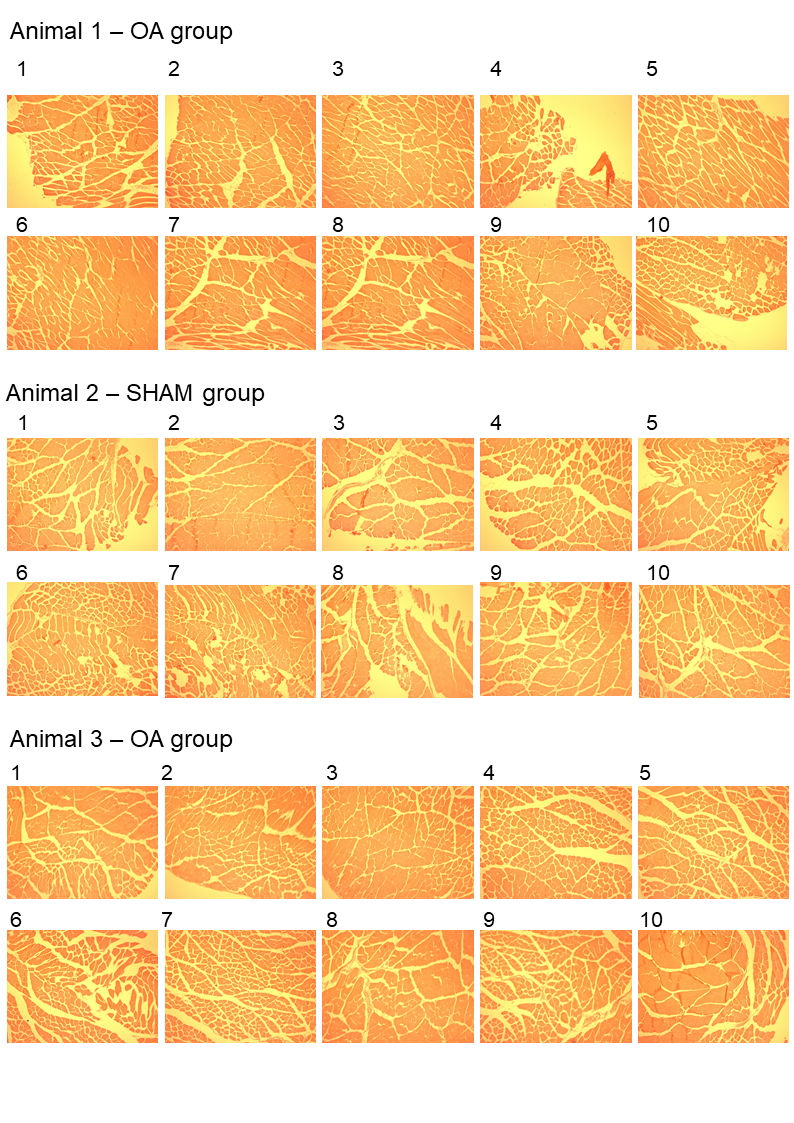

Supplement: S3 Fig — Samples of right hind paw gastrocnemius stained with hematoxylin-eosin of OA and SHAM animals at the end of the experimental period. Magnification: × 200. (TIF) [file pone.0196682.s003.tif]

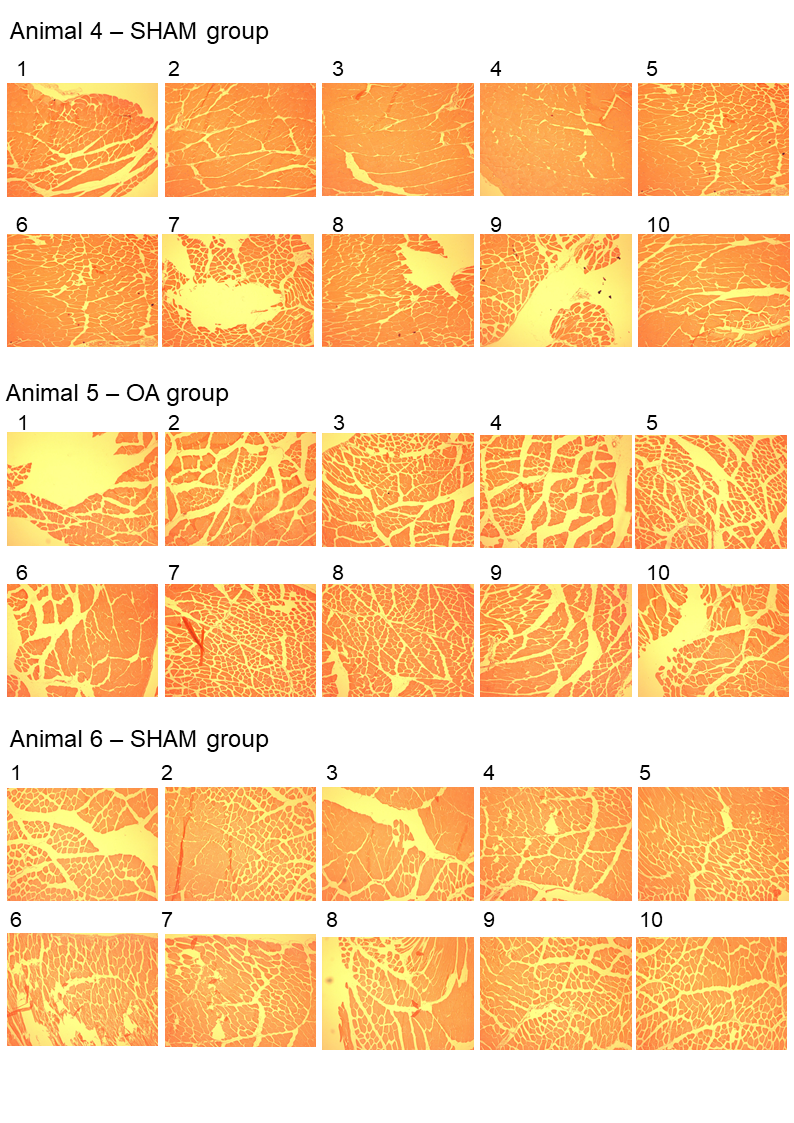

Supplement: S4 Fig — Samples of right hind paw gastrocnemius stained with hematoxylin-eosin of OA and SHAM animals at the end of the experimental period. Magnification: × 200. (TIF) [file pone.0196682.s004.tif]

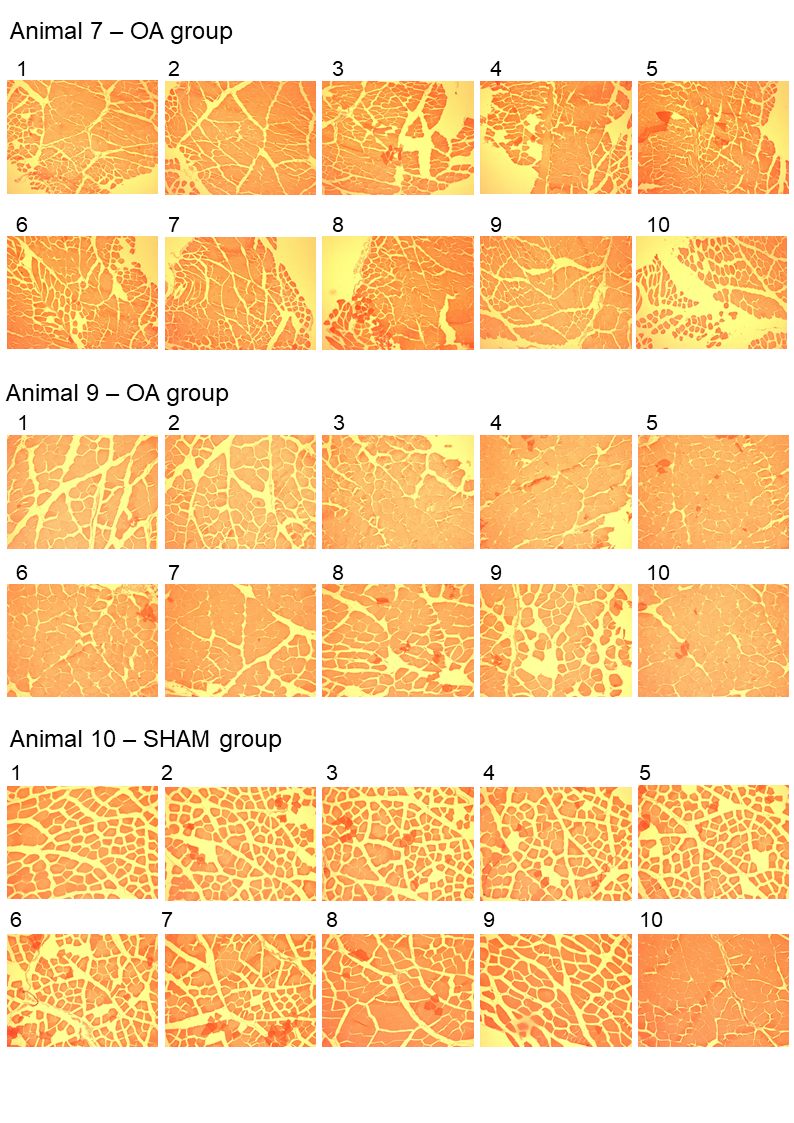

Supplement: S5 Fig — Samples of right hind paw gastrocnemius stained with hematoxylin-eosin of OA and SHAM animals at the end of the experimental period. Magnification: × 200. (TIF) [file pone.0196682.s005.tif]

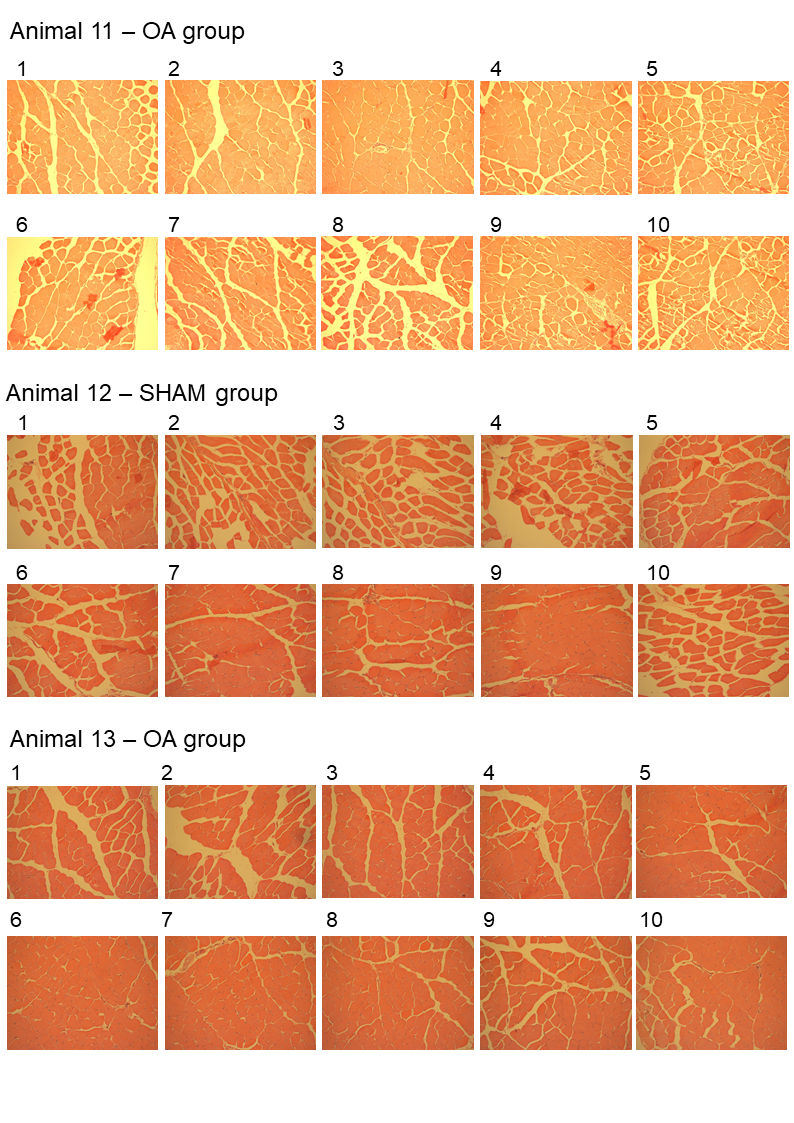

Supplement: S6 Fig — Samples of right hind paw gastrocnemius stained with hematoxylin-eosin of OA and SHAM animals at the end of the experimental period. Magnification: × 200. (TIF) [file pone.0196682.s006.tif]

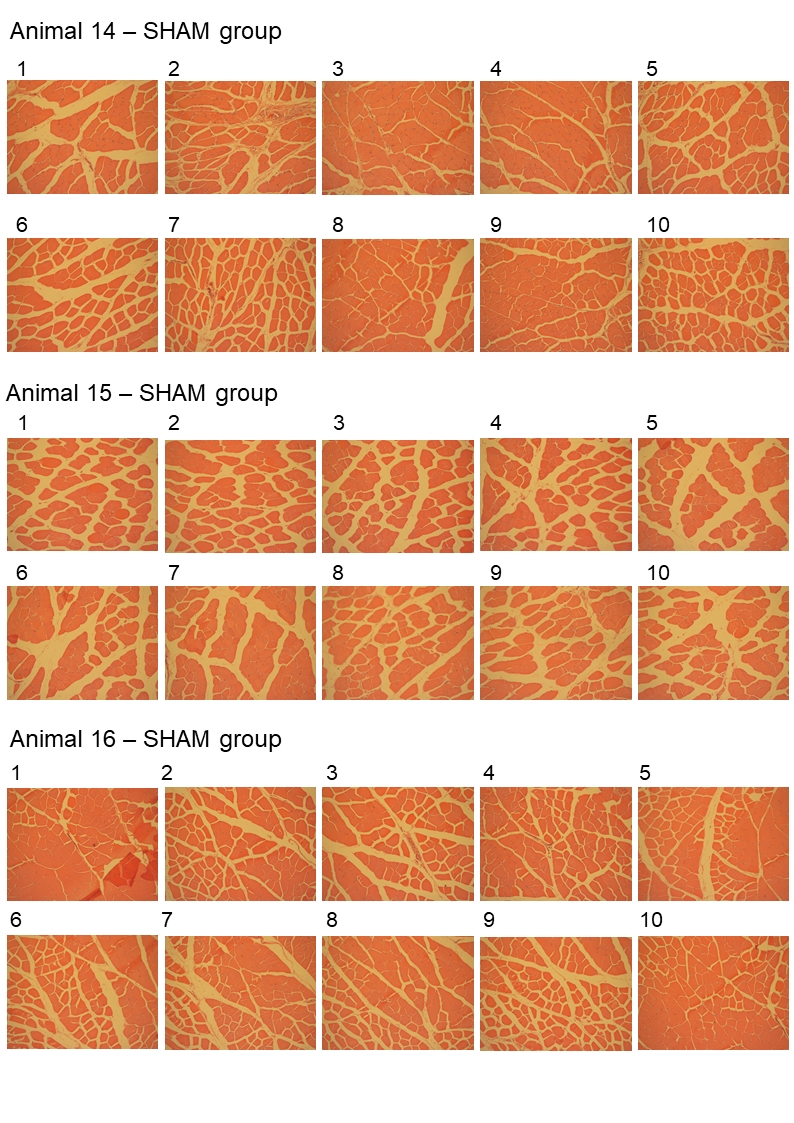

Supplement: S7 Fig — Samples of right hind paw gastrocnemius stained with hematoxylin-eosin of OA and SHAM animals at the end of the experimental period. Magnification: × 200. (TIF) [file pone.0196682.s007.tif]

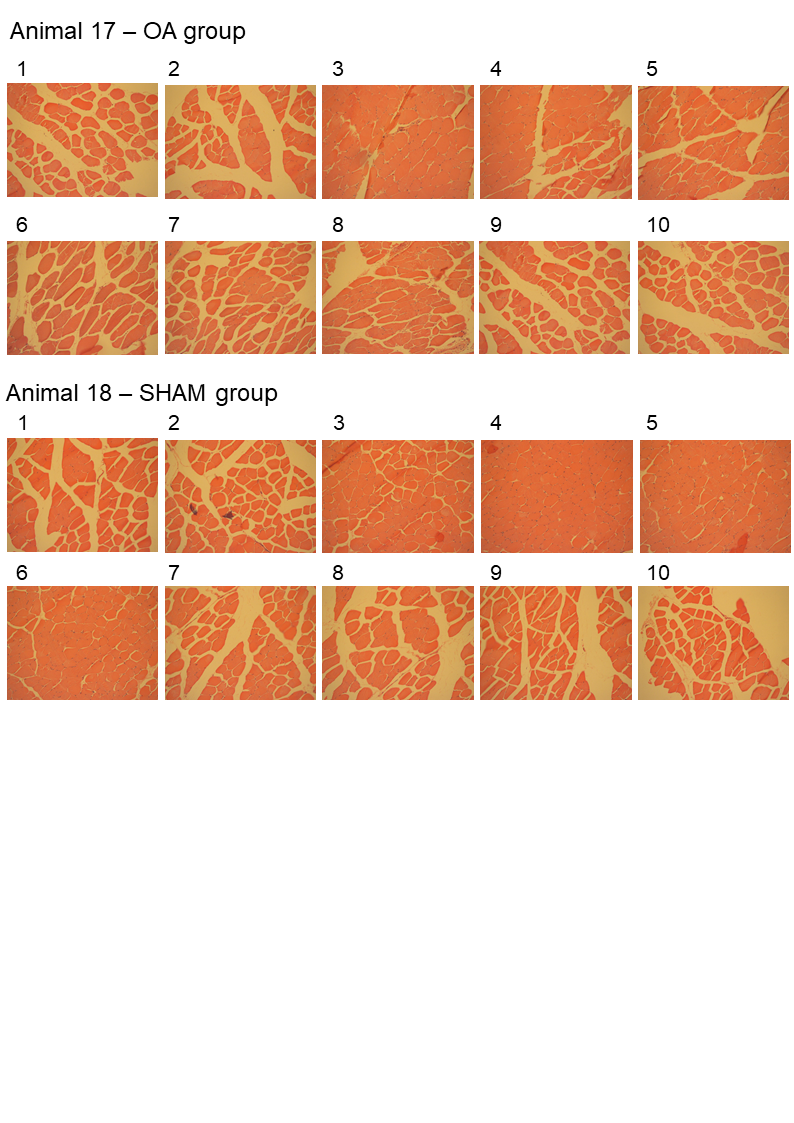

Supplement: S8 Fig — Samples of right hind paw gastrocnemius stained with hematoxylin-eosin of OA and SHAM animals at the end of the experimental period. Magnification: × 200. (TIF) [file pone.0196682.s008.tif]

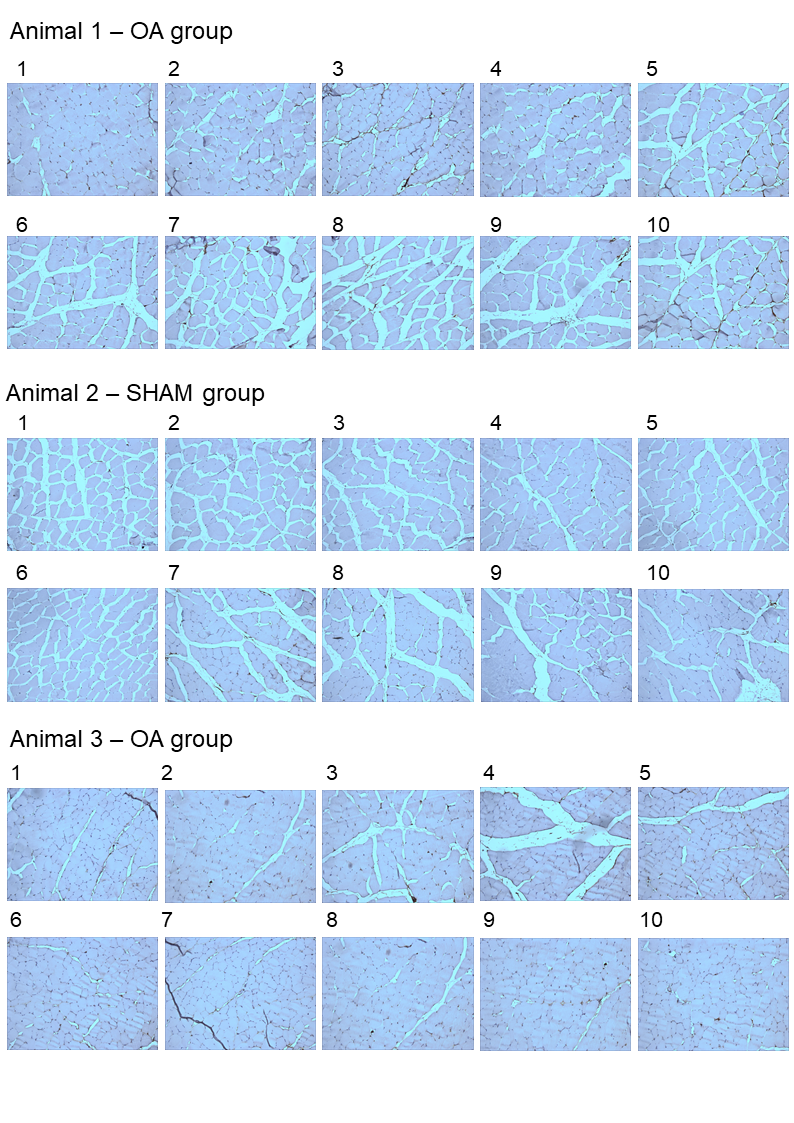

Supplement: S9 Fig — Samples of right hind paw gastrocnemius of OA and SHAM animals stained for IL-1β, at the end of the experimental period. Magnification: × 200. (TIF) [file pone.0196682.s009.tif]

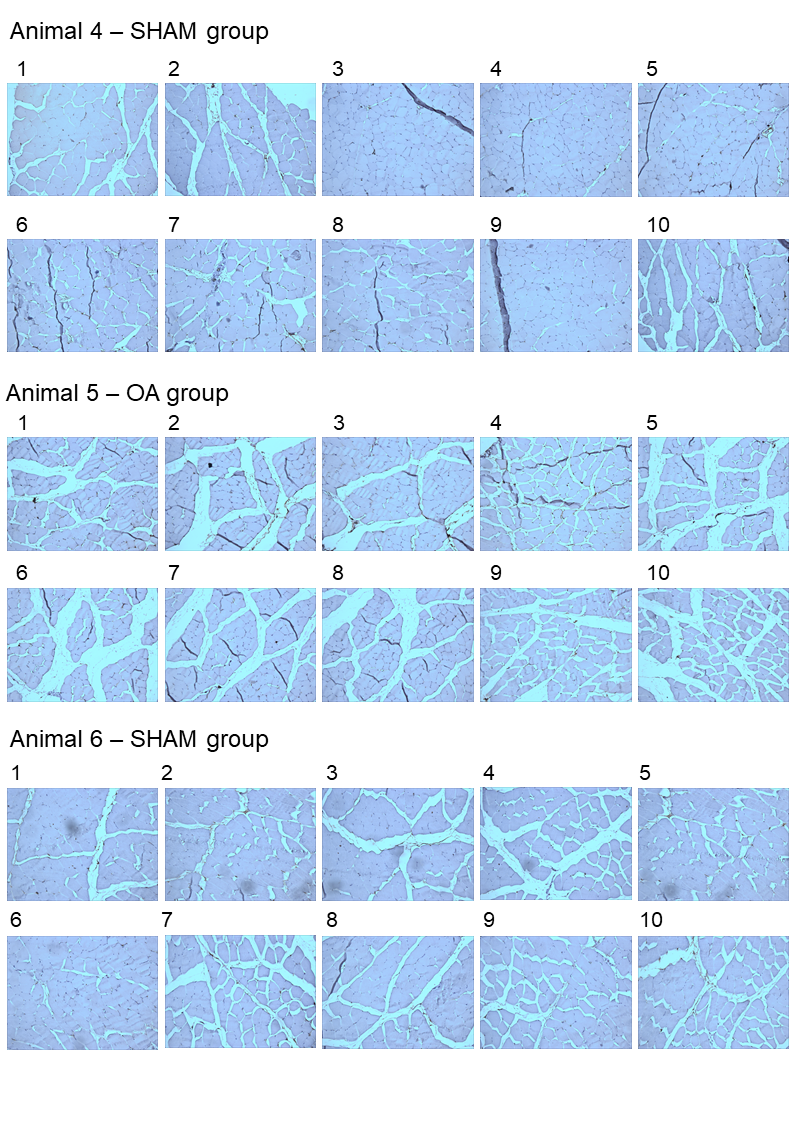

Supplement: S10 Fig — Samples of right hind paw gastrocnemius of OA and SHAM animals stained for IL-1β, at the end of the experimental period. Magnification: × 200. (TIF) [file pone.0196682.s010.tif]

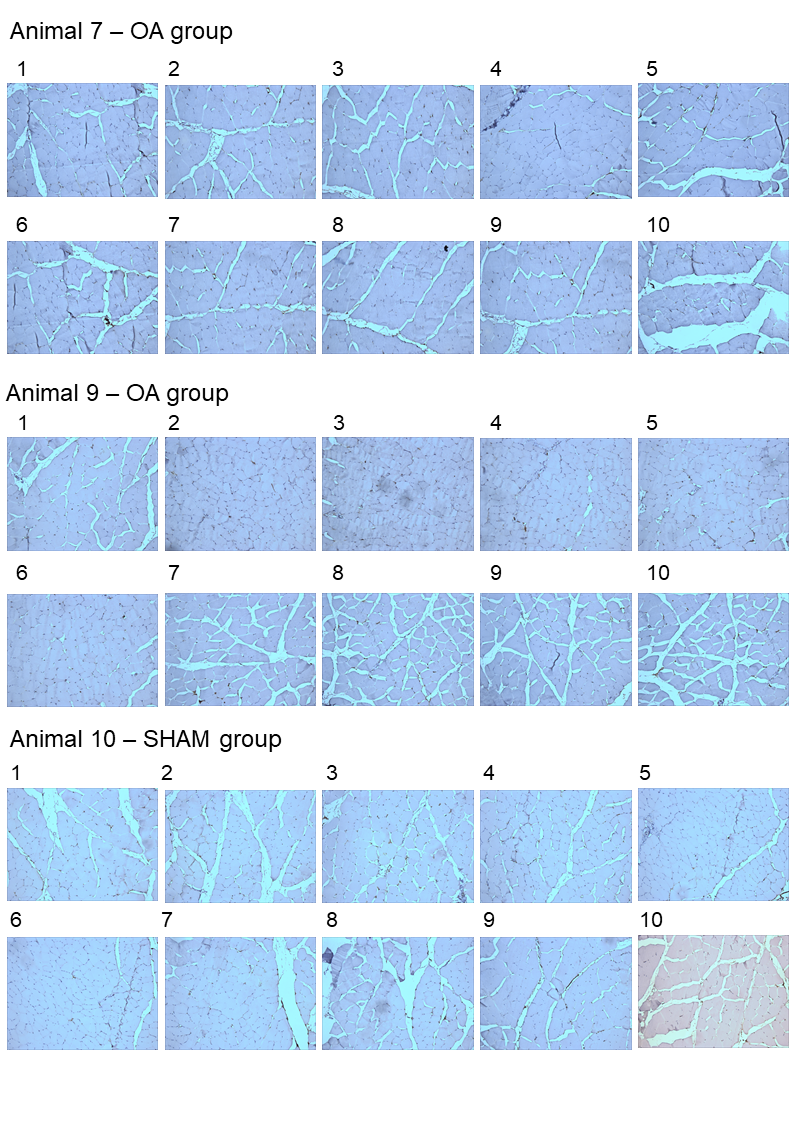

Supplement: S11 Fig — Samples of right hind paw gastrocnemius of OA and SHAM animals stained for IL-1β, at the end of the experimental period. Magnification: × 200. (TIF) [file pone.0196682.s011.tif]

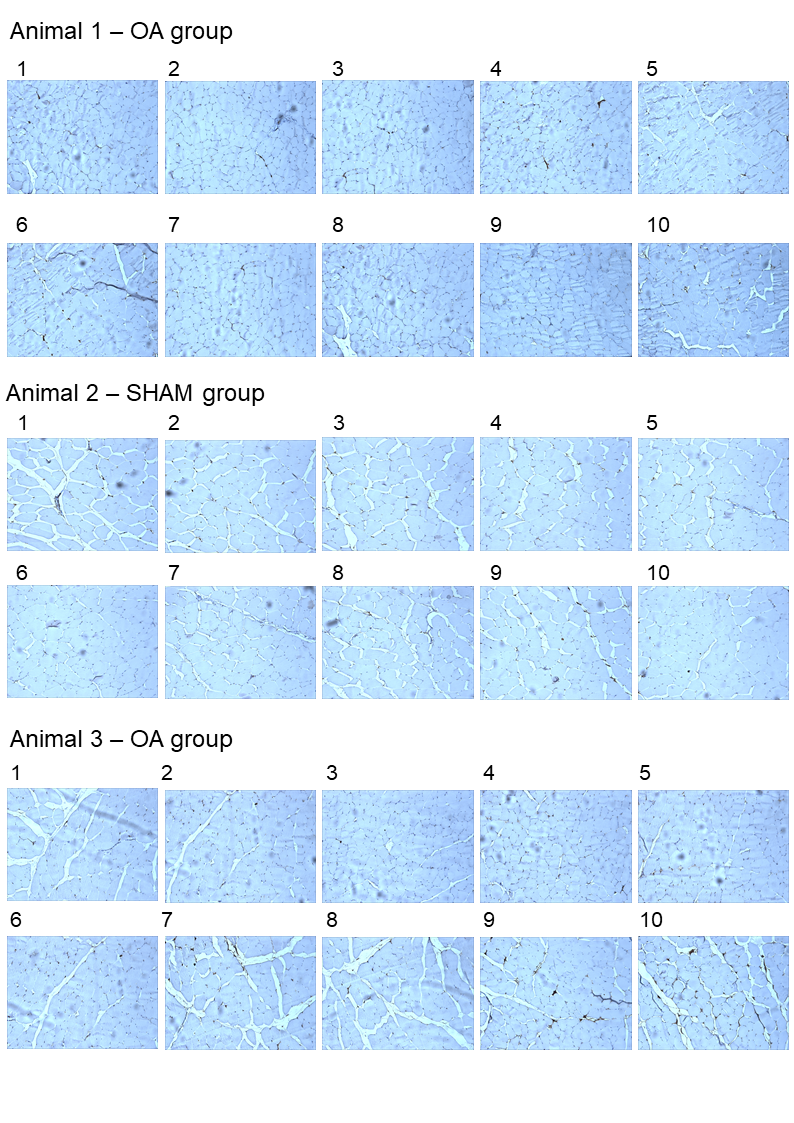

Supplement: S12 Fig — Samples of right hind paw gastrocnemius of OA and SHAM animals stained for TNF-α, at the end of the experimental period. Magnification: × 200. (TIF) [file pone.0196682.s012.tif]

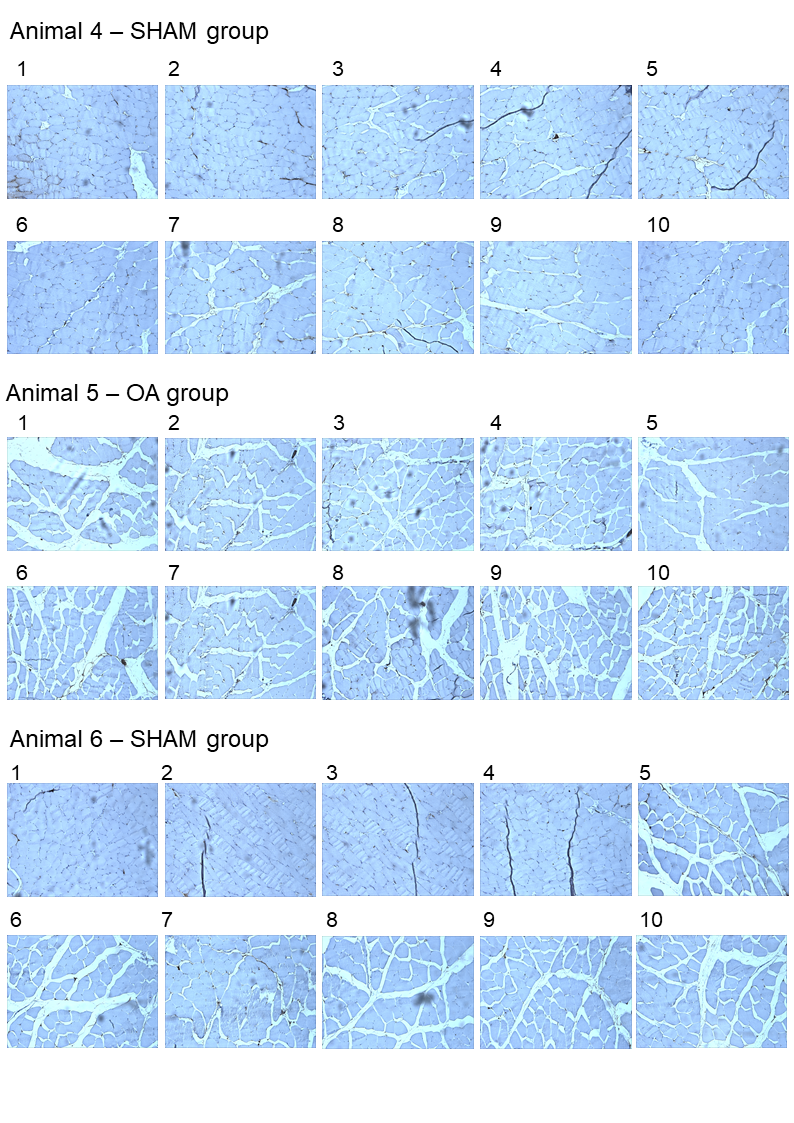

Supplement: S13 Fig — Samples of right hind paw gastrocnemius of OA and SHAM animals stained for TNF-α, at the end of the experimental period. Magnification: × 200. (TIF) [file pone.0196682.s013.tif]

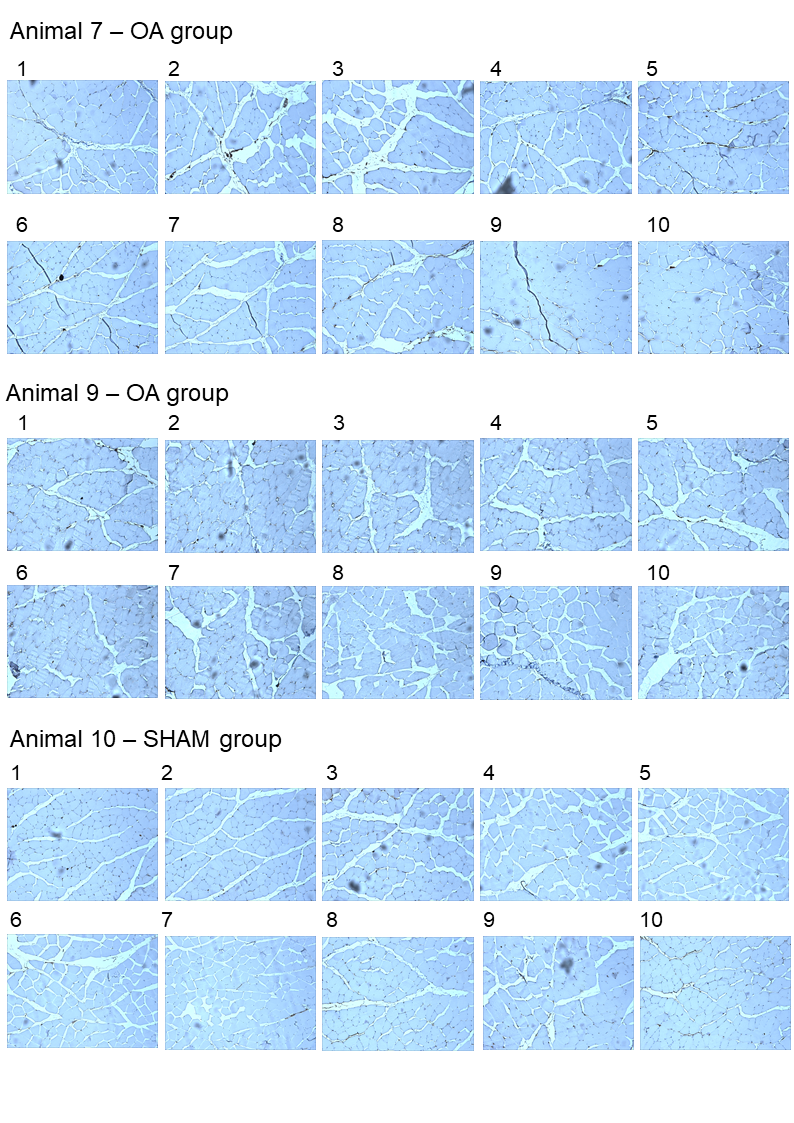

Supplement: S14 Fig — Samples of right hind paw gastrocnemius of OA and SHAM animals stained for TNF-α, at the end of the experimental period. Magnification: × 200. (TIF) [file pone.0196682.s014.tif]

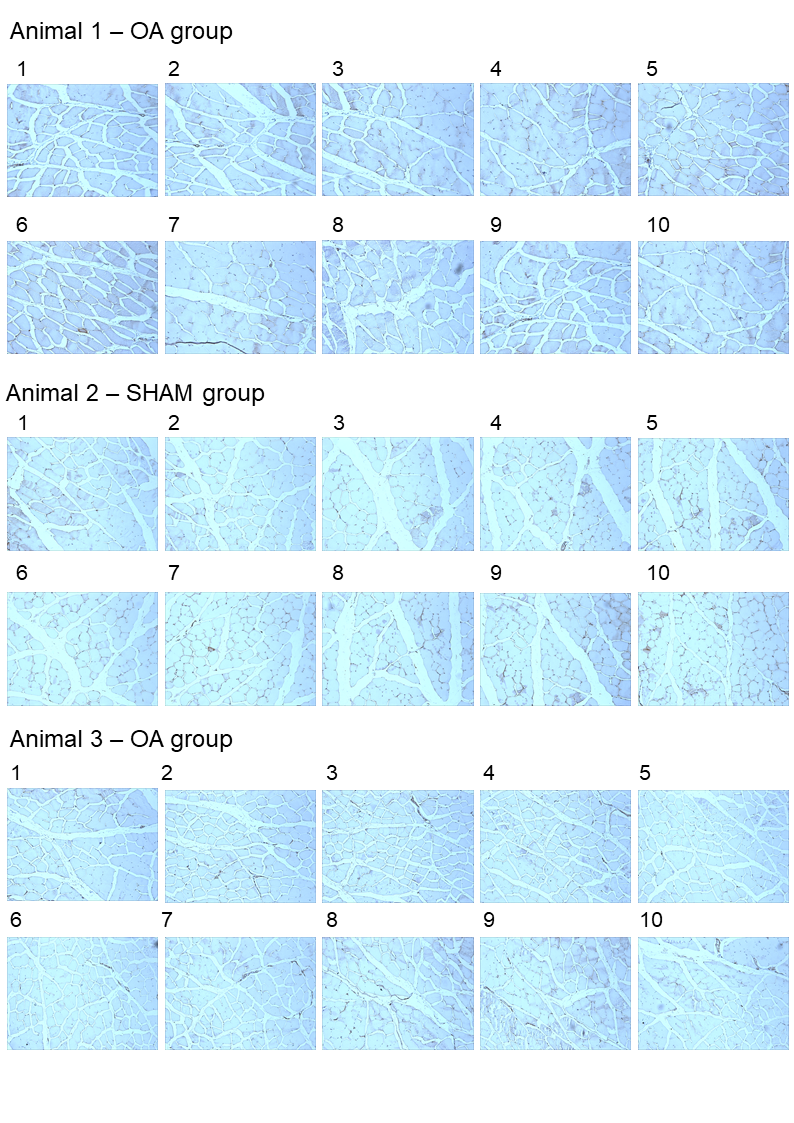

Supplement: S15 Fig — Samples of right hind paw gastrocnemius of OA and SHAM animals stained for Pax7, at the end of the experimental period. Magnification: × 200. (TIF) [file pone.0196682.s015.tif]

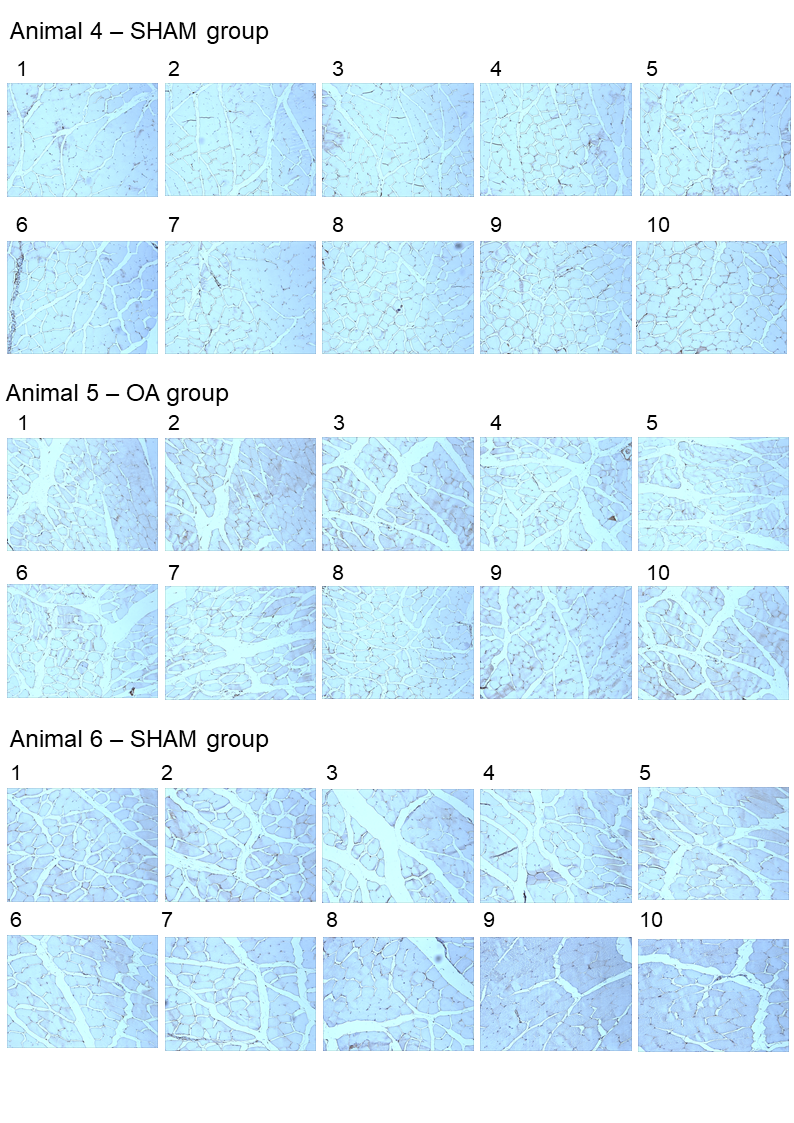

Supplement: S16 Fig — Samples of right hind paw gastrocnemius of OA and SHAM animals stained for Pax7, at the end of the experimental period. Magnification: × 200. (TIF) [file pone.0196682.s016.tif]

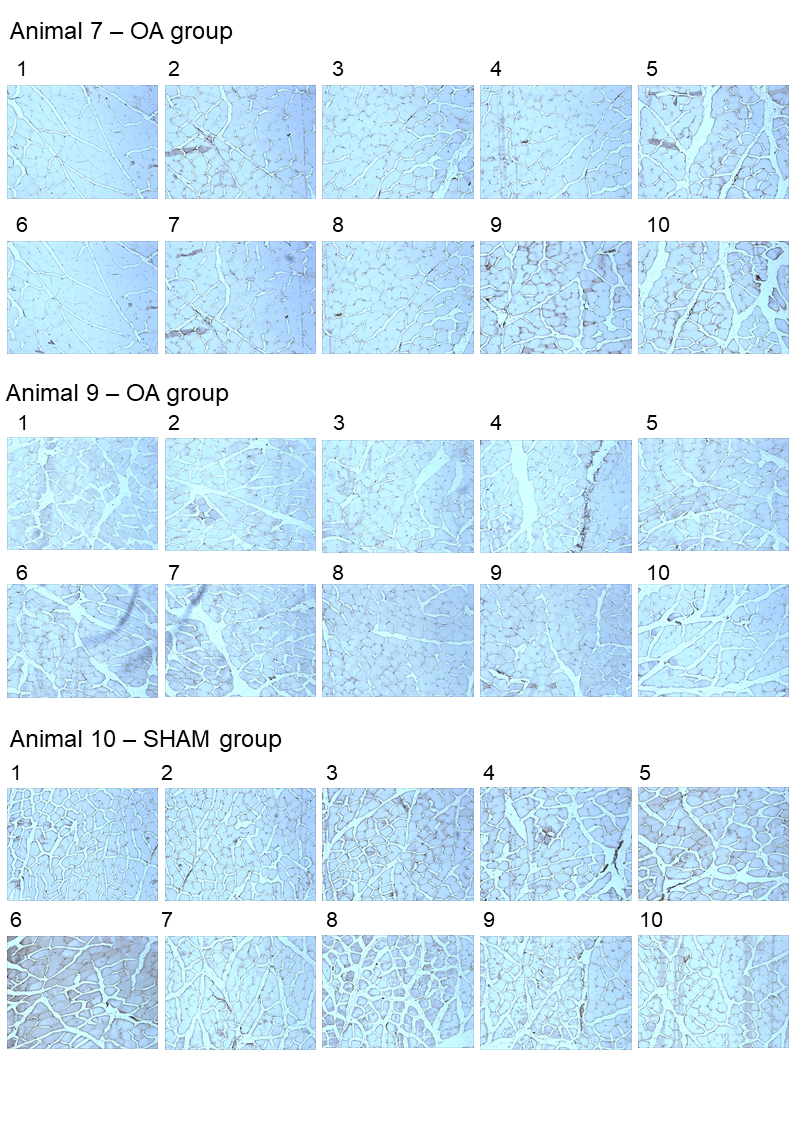

Supplement: S17 Fig — Samples of right hind paw gastrocnemius of OA and SHAM animals stained for Pax7, at the end of the experimental period. Magnification: × 200. (TIF) [file pone.0196682.s017.tif]
